# Hypersensitivity relief of MIH-affected molars using two sealing techniques: a 12-week follow-up

**DOI:** 10.1007/s00784-021-04163-5

**Published:** 2021-09-01

**Authors:** Katrin Bekes, Stefanie Amend, Julia Priller, Claudia Zamek, Tanja Stamm, Norbert Krämer

**Affiliations:** 1 Department of Paediatric Dentistry, University Clinic of Dentistry, Medical University of Vienna, Sensengasse 2a, 1090 Vienna, Austria; 2 Department of Paediatric Dentistry, Medical Centre for Dentistry Section for Outcomes Research, University Medical Center Giessen and Marburg, Campus Giessen, Schlangenzahl 14, 35392 Giessen, Germany; 3 Private Practice, Graf-Adolf-Straße 24, 40212 Düsseldorf, Germany; 4 Section for Outcomes Research, Center for Medical Statistics, Informatics, and Intelligent Systems, Medical University of Vienna, Spitalgasse 23, 1090 Vienna, Austria

**Keywords:** Molar incisor hypomineralization (MIH), Hypersensitivity, Sealing, Composite, Glass ionomer cement

## Abstract

**Objectives:**

The aim of this study was to compare the efficacy in reducing hypersensitivity in molar incisor hypomineralization (MIH)-affected molars immediately and over 12 weeks after sealing using two different materials (composite and glass ionomer). Furthermore, the retention rates of both materials were analyzed.

**Methods:**

Thirty-nine children with two MIH-affected molars showing hypersensitivity and non-occlusal breakdowns were included. Hypersensitivity was assessed with an evaporative (air) stimulus. Both teeth were sealed by two calibrated operators using a split-mouth design with either Clinpro Sealant in combination with Scotchbond Universal (C) or Ketac Universal (K), respectively. Clinical pain assessments (Schiff Score Air Sensitivity Scale [SCASS], Visual Analog Scale [VAS]) were made at baseline (“pre”), immediately after treatment (“post”), and after 1, 4, 8, and 12 weeks. Paired *t* tests were calculated in each group between baseline and all other time points.

**Results:**

Thirty-eight children with 76 molars completed all stages of the study. Regardless of the material used, the application of the sealant decreased hypersensitivity significantly immediately as well as throughout the 12-week recalls (all *p* values < 0.001). We found no statistically significant difference among both materials chosen in any of the time points evaluated. Furthermore, retention of both materials was comparable in both groups.

**Conclusions:**

Both sealant materials were able to reduce hypersensitivity successfully immediately and throughout the 12-week follow-up. Furthermore, their performance was similar in terms of retention.

**Clinical relevance:**

Hypersensitivity can be a major complaint in patients with MIH. This is the first study evaluating hypersensitivity relief of MIH-affected molars using two sealing techniques.

## Introduction

Molar incisor hypomineralization (MIH) is defined as “demarcated, qualitative developmental defects of systemic origin of the enamel of one or more first permanent molars with or without the involvement of incisors” [1]. Recent data indicates that MIH is a frequently encountered dental condition worldwide [2]. Although MIH is considered to be an idiopathic condition, its concise etiology remains unclear [3]. Clinically, affected teeth show a hypomineralization which can be seen as an alteration in the translucency of the enamel. Hypomineralized enamel can vary in color shade from white to yellow or brown [4], but always shows borders that are well-defined and distinct from sound enamel [5]. Depending on the severity, the condition could be associated with dental complications including rapid wear, enamel loss, increased susceptibility to caries, loss of fillings, and most of all, severe hypersensitivity often resulting in severe discomfort. At dental examination, behavior management problems and even dental fear are common [6]. With respect to hypersensitivity, children often report that hot and cold or sweet drinks and meals, toothbrushing, and even airflow cause pain [1, 6, 7]. The reason for hypersensitivity is still not fully understood [8]. Rodd et al. hypothesized that the high porosity of the hypomineralized enamel favors the penetration of bacteria in the dentinal tubules, causing a subclinical pulpal inflammation [9]. Furthermore, clinical studies regarding the prevalence or intensity of dental hypersensitivity in teeth affected by MIH that had been conducted in a standardized way are limited [10].

The management of MIH is challenging due to the widely varying severity, with a broad spectrum of treatment modalities being available, ranging from prevention of enamel breakdown or caries, management of hypersensitivity or pain, restorative treatments, to extraction with or without subsequent orthodontic treatment [11].

Definitely, it is very important to start an enhanced preventive program as soon as an MIH-affected tooth erupts [11, 12]. The preventive approach includes thorough oral hygiene with fluoride toothpaste as well as the application of other topical fluoride varnishes with regard to caries risk. Casein phosphopeptide-amorphous calcium phosphate (CPP-ACP) oral care products are likewise suggested for remineralization and desensitization [13, 14]. For example, Pasini et al. compared the use of CPP-ACP to usual oral care (fluoride toothpaste) and found it to be superior in terms of reduction of tooth sensitivity to both thermal and mechanical stimuli [14]. Furthermore, Bekes et al. proposed arginine-containing products for the treatment of MIH-affected teeth to reduce the associated hypersensitivity [15]. However, regarding desensitizing treatment, still very limited data are currently available.

In addition to these mentioned preventive approaches, sealing of MIH molars is considered a valuable and effective preventive measure [12]. Generally, pit and fissure sealants are one of the most highly recommended and widely accepted dental procedures for the prevention or control of caries on occlusal surfaces [16]. Their effectiveness has been documented in numerous clinical studies. A Cochrane review found that sealants placed on the occlusal surfaces of permanent molars in children reduced caries up to 48 months when compared to the no sealant control [16]. Furthermore, a meta-analysis was able to show that the overall effectiveness of autopolymerized fissure sealants in preventing dental decay was 71% [17]. Regarding MIH, there is conflicting evidence on the success rate of resin-based sealants on MIH-affected molars compared to sound molars. Lygidakis et al. proposed the use of a single bottled adhesive prior to sealant application to increase substantially the retention rates of the sealants in these molars [18]. As MIH-affected children also show a decreased quality of life [19] and an increased risk of developing behavior management problems [7], simplified sealing procedures are preferable. For example, adhesively bonded sealants avoid conventional acid etching and decrease the technique sensitivity and application time may also result in increased patient comfort during the placement of the material, which is especially important in pediatric dentistry [20].

Until now, there are no data available if sealants might have an influence on hypersensitivity relief in MIH molars as well. Merely, Linner et al. [21] described the covering of hypersensitive occlusal surfaces in these teeth. Thus, the objective of this study was to compare the efficacy in reducing hypersensitivity in MIH-affected molars immediately and over 12 weeks after sealing using two different materials (composite and glass ionomer). Furthermore, it was the aim to compare the retention rates of both materials used over these weeks.

## Materials and methods

### Subjects and setting

For this 12-week prospective two-center study, patients with MIH were recruited from the Department for Paediatric Dentistry of the University Clinic of Dentistry in Vienna, Austria, and a private practice in Düsseldorf, Germany. For the diagnosis of MIH, the criteria suggested by the European Academy of Paediatric Dentistry (EAPD) [22] were used. The inclusion criteria were children aged 6–10 with two hypersensitive MIH-affected first permanent molars which had a qualifying response to an air blast stimulus applied for 1 s as defined by a score of two or three on the Schiff Cold Air Sensitivity Scale (SCASS) [23], no breakdowns (MIH-TNI 3) [24, 25], and opacities to be at least present at least on the occlusal surfaces.

All dentists at both study centers were briefed about the details of the study and asked to recruit patients. One calibrated dentist in Austria (KB) and in Germany (CZ) examined potential MIH patients for inclusion into the study. In each case, they confirmed the diagnosis of MIH and determined the presence of hypersensitivity. Promising hypomineralized molars were selected in response to an air blast stimulus. The air was delivered from a standard dental unit air syringe for 1 s at a distance of 1 cm and perpendicular to the occlusal surface of the tooth. Neighboring teeth were shielded with cotton rolls or with the fingers of the examiner. The Schiff Cold Air Sensitivity Scale (SCASS) was used to assess subject response to this stimulus (0=no response to the stimulus; 1=no response to the stimulus, patient considers stimulus to be painful; 2= response to stimulus, patient moves from the stimulus; 3= response to the stimulus, patient moves from the stimulus and requests immediate discontinuation of the stimulus) [23].

In each patient, both hypersensitive MIH teeth were sealed with Clinpro Sealant in combination with Scotchbond Universal or Ketac Universal (3M, Seefeld, Germany) (Table 1), using a split-mouth design. Randomization was performed with Excel (Microsoft, Redmond, USA). Clinpro Sealant represented treatment A, and Ketac represented treatment B. The two teeth that were chosen for the sealing were sorted by their quadrants.

**Table 1:** Compositions of the materials used

Material	Composition
Scotchbond™ Universal	2-Hydroxyethyl methacrylate; bisphenol a diglycidyl ether dimethacrylate (BISGMA), 2-propenoic acid, 2-methyl-, reaction products with 1,10-decanediol and phosphorous oxide (P2O5); ethanol; water; 2-propenoic acid, 2-methyl-, 3-(trimethoxysilyl)propyl ester with vitreous silica; copolymer of acrylic and itaconic acid; camphorquinone dimethylaminobenzoate(-4); (dimethylamino)ethyl methacrylate
Clinpro™ Sealant	Triethylene glycol dimethacrylate (TEGDMA); bisphenol a diglycidyl ether dimethacrylate (BISGMA); silane-treated silica; tetrabutylammonium tetrafluoroborate; diphenyliodonium hexafluorophosphate; triphenylantimony; ethyl 4-dimethyl aminobenzoate (EDMAB); titanium dioxide hydroquinone
Ketac™ Universal Aplicap™	Liquid: water; copolymer of acrylic acid-maleic acid; tartaric acid; benzoic acid Powder: oxide glass chemicals

Clinpro Sealant is a light-cure, resin-based sealant, Scotchbond Universal is a one-component, light-curing adhesive, and Ketac Universal is a radiopaque glass ionomer cement. All sealings were placed without using analgesic measures. Before sealing, both teeth were cleaned with Clinpro Prophy Paste (3M, Seefeld, Germany) using a bristle brush. Cotton rolls and a four-hand technique were applied for isolation. In the resin-based sealant group, Scotchbond Universal was rubbed in the cleaned fissure for 20 s and air dried for 5 s. Afterwards, Clinpro Sealant was applied with a syringe. Before light-curing, the presence of air bubbles was checked and teased out. In the glass ionomer cement group, Ketac Universal was applied after activating and mixing the capsule. Subsequently, it was covered with Ketac Bond (3M, Seefeld, Germany) and cured. Occlusion control was performed in using articulating paper. If necessary, adjustments with finishing burs were made. For the duration of the study, patients were instructed to use the toothpaste given to them (Clin Pro Tooth Crème, 3M, Seefeld, Germany).

The enrolment into this study was voluntary. The patients’ legal representatives were informed about the study procedures in written and verbal forms. The study was approved by the Ethics Committee of the Medical University of Vienna and the University of Giessen.

Sample size calculation was performed regarding the reduction of hypersensitivity after 12 weeks using two sealants. Based on previous studies, a reduction of hypersensitivity by two points on the scale of 0–3 was assumed to be clinically relevant [15]. With a required power of 95% and a significance of 5%, 47 individuals were required. The plan was to conservatively enroll at least 52 participants by taking an equal number of patients from each center.

### Data collection

All participants were evaluated before and immediately after treatment and after 1, 4, 8, and 12 weeks. Air blast hypersensitivity examinations were performed at each time point using the Schiff Score (SCASS) as described above. In addition, children were asked to rank the pain intensity after the air stimulus was applied to the MIH molar with the Wong-Baker Faces Scale (WBFS) [26]. Thereby, the children subjectively judged their pain perception by using a display (smiling face = scale 0: no pain; crying face = scale 10: hurts most).

**Fig. 1:**
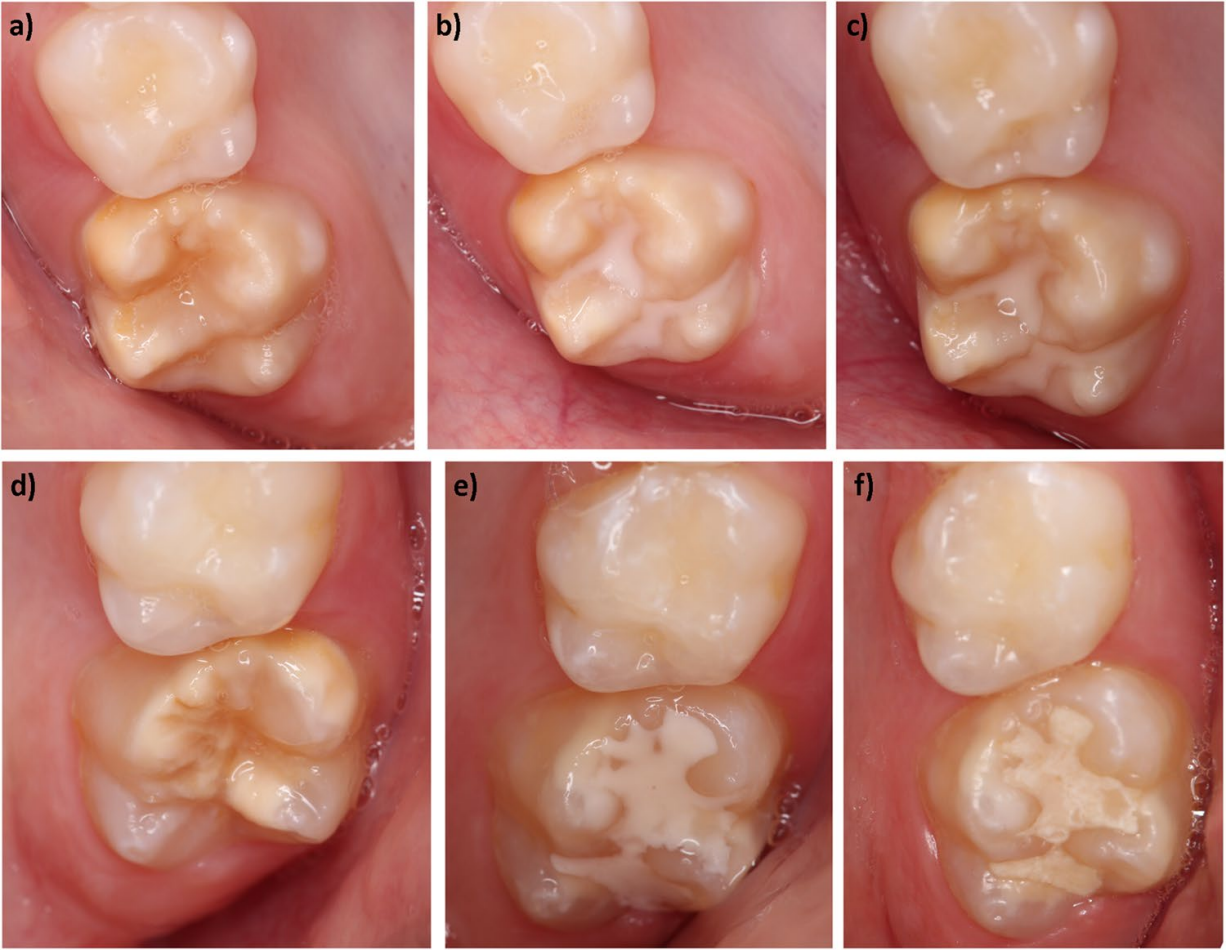
Sealings in a patient in the upper jaw at baseline and after 12 weeks. **a** Tooth 16 at baseline. **b** tooth 16 after sealing with Clinpro™ Sealant. **c** tooth 16 after 12 weeks. **d** tooth 26 at baseline. **d** tooth 26 after sealing with Ketac™ Universal. **e** tooth 26 after 12 weeks

For the secondary objective of the study, the retention rates as well as marginal integrity and discoloration, surface texture (optical and tactile), and presence of a carious lesion were recorded at the follow-up time points after 1, 4, 8, and 12 weeks. The clinical assessments were based on the modified USPHS criteria and criteria proposed by Dukic [27–29] (Table 2). Furthermore, a photographic documentation was made at each follow-up examination (Fig. 1).

**Table 2:** Criteria used for the evaluation of the fissure sealants

Category	Scores	Criteria
Retention	Alpha Bravo Charlie	No loss of sealing material Partial loss of sealing material Loss of restorative material
Marginal adaption	Alpha Bravo Charlie	Closely adapted, no visible crevice Visible crevice, explorer will penetrate Crevice in which dentin is exposed
Marginal discoloration	Alpha Bravo Charlie	No discoloration Discoloration without penetration in pulpal direction Discoloration with penetration in pulpal direction
Surface texture—optical	Alpha Bravo	Shiny Dull
Surface texture—tactile	Alpha Bravo Charlie	Enamel-like surface Surface rougher than enamel, clinically acceptable Surface unacceptably rough
Caries	Alpha Charlie	No caries present Caries present

Baseline and follow-up examinations were done by one dentist at each study center (KB, CZ). The photographs taken at each time point were also evaluated by a second examiner at each center (JP, SA). Disagreements between the investigators were resolved by consensus. Blinding at baseline and at follow-ups was not possible due to different application protocols and different materials used.

### Data analysis

We first summarized the data descriptively. We calculated mean values and standard deviations, as well as absolute and relative frequencies, where appropriate. SCASS and VAS scores were compared in a pairwise analysis between two time points using *t* tests. Associations between the independent variable (group) and the dependent variable (clinical evaluation score) were assessed using Fisher’s exact test at a 5% level of significance. The analysis was performed using the statistical program SPSS 26.0 (IBM, Chicago, USA).

## Results

Over 2 years, it was possible to enroll thirty-nine patients in both study centers (20 patients from Austria and 19 patients from Germany). Included children had a mean age of 7.5 years (SD = 1.3, age range from 6 to 10 years). Of these, 18 (46.2 %) were female. The patients had a mean dmft of 0.38 (±1.44) and a mean DMFT of 0.21 (±0.41), respectively (Table 3). Before treatment, included MIH patients showed two affected molars with high-intensity hypersensitivity (SCASS ≥2). One child did not show up at all follow-ups and was therefore excluded for hypersensitivity analysis.

**Table 3:** General characteristics of participants (*N*=39)

Patient variable	*n* (%) mean (SD; range)
Age (years)	7.5 (1.3, 6–10)
Gender	
Male	21 (53.8)
Female	18 (46.2)
Caries index	
dmft	0.38 (±1.44; 0–8)
DMFT	0.21 (±0.41; 0–1)

Table 4 shows the mean scores and standard deviations for the air blast test (Schiff score and Wong-Baker Faces Scale) at different time points. In the resin-based sealant group (Clinpro Sealant with Scotchbond), the SCASS was 2.3 (±0.5; range = 2–3) and the WBFS score was 7.0 (±1.8; range = 2–10). In the glass ionomer cement group (Ketac Universal), both baseline scores were similar (SCASS 2.4 [±0.5; range = 2–3]; WBFS score 7.0 [±2.0; range = 2–10]) (Table 3). Regardless of the material used, there was a significant difference in hypersensitivity in all 38 patients immediately and at all further follow-up times after sealing measured with the SCASS and the WBFS score (*T* test, *p* < 0.01) (Table 5). The mean SCASS scores decreased significantly following treatment to 0.4 in both groups revealing a large effect size (Table 3). The WBFS scores were also significantly reduced to 1.2 (±0.5; Clinpro) and 1.1 (±1.8; Ketac), respectively (Table 4). Pairwise comparison of the post-measurement time points showed no significant differences.

**Table 4:** Mean scores and standard deviations for the air blast test (Schiff score) and the tactile test (Wong-Baker Faces Scale) at different time points

*N* = 38	SCASS		VAS	
	Clinpro	Ketac	Clinpro	Ketac
Before treatment (pre)	2.3 (±0.5)	2.4 (±0.5)	7.1 (±1.7)	7.1 (±2.0)
After treatment (post)	0.4 (±0.6)	0.4 (±0.7)	1.2 (±1.7)	1.1 (±1.8)
After 1 week	0.3 (±0.7)	0.3 (±0.7)	1.2 (±1.9)	1.1 (±1.8)
After 4 weeks	0.2 (±0.6)	0.3 (±0.7)	0.8 (±1.3)	1.1 (±1.9)
After 8 weeks	0.2 (±0.5)	0.3 (±0.7)	0.7 (±1.2)	0.7 (±1.5)
After 12 weeks	0.1 (±0.4)	0.1 (±0.5)	0.8 (±1.4)	0.8 (±1.3)

**Table 5:** Changes in SCASS and VAS and related effect sizes at different time points

	Clinpro				Ketac			
	*p* value	Change score mean (SD)	Effect size	Description	*p* value	Change score mean (SD)	Effect size	Description
**SCASS**								
Baseline								
Immediately post 1 week post	<0.001 <0.001	1.9 (±0.8) 2.1 (±0.8)	2.4 2.6	Large Large	<0.001 <0.001	2.0 (±0.7) 2.0 (±0.9)	2.9 2.2	Large Large
4 weeks post	<0.001	2.1 (±0.8)	2.6	Large	<0.001	2.0 (±0.8)	2.5	Large
8 weeks post	<0.001	2.2 (±0.7)	3.1	Large	<0.001	2.1 (±0.7)	3	Large
12 weeks post	<0.001	2.2 (±0.6)	3.7	Large	<0.001	2.2 (±0.6)	3.7	Large
Immediately post								
1 week post 4 weeks post	=0.324 =0.147	0.1 (±0.6) 0.2 (±0.7)	0.2 0.3	Small Small	=0.661 =0.700	0.1 (±0.7) 0.1 (±0.8)	0.1 0.1	Small
8 weeks post	=0.088	0.2 (±0.7)	0.3	Small	=0.244	0.2 (±0.8)	0.3	Small
12 weeks post	=0.010	0.3 (±0.7)	0.4	Moderate	=0.023	0.3 (±0.7)	0.4	Moderate
1 week post								
4 weeks post	=0.597	0.1 (±0.9)	0.1		=1	0 (±1.0)		
8 weeks post	=0.457	0.1 (±0.9)	0.1		=0.457	0.1 (±0.9)	0.1	
12 weeks post	=0.090	0.2 (±0.7)	0.3	Small	=0.088	0.2 (±0.7)	0.3	Small
4 weeks post								
8 weeks post	=0.324	0 (±0.2)			=0.291	0.1 (±0.6)	0.2	Small
12 weeks post	=0.378	0.1 (±0.7)	0.1		=0.103	0.2 (±0.8)	0.3	Small
8 weeks post								
12 weeks post	=0.474	0.1 (±0.7)	0.1		=0.401	0.1 (±0.8)	0.1	
**VAS**								
Baseline								
immediately post 1 week post	<0.001 <0.001	5.8 (±2.4) 5.8 (±2.5)	2.4 2.3	Large Large	<0.001 <0.001	6.0 (±2.3) 5.9 (±2.0)	2.6 3.0	Large Large
4 weeks post	<0.001	6.3 (±2.2)	2.9	Large	<0.001	6.1 (±2.5)	2.4	Large
8 weeks post	<0.001	6.4 (±2.2)	2.9	Large	<0.001	6.4 (±2.2)	2.9	Large
12 weeks post	<0.001	6.2 (±2.0)	3.1	Large	<0.001	6.2 (±2.1)	3.0	Large
Immediately post								
1 week post 4 weeks post	=0.793 =0.136	−0.1 (±1.2) 0.5 (±1.9)	0.1 0.3	Small Small	=0.918 =0.810	0 (±1.6) 0.1 (±2.0)	0.1 0.1	Small
8 weeks post	=0.085	0.6 (±1.9)	0.3	Small	=0.265	0.4 (±2.1)	0.2	Small
12 weeks post	=0.156	0.4 (±1.6)	0.3	Small	=0.398	0.3 (±1.9)	0.2	Small
1 week post								
4 weeks post	=0.082	0.5 (±1.8)	0.3	Small	=0.783	0.1 (±2.3)	0	
8 weeks post	=0.069	0.6 (±2.0)	0.3	Small	=0.280	0.4 (±2.4)	0.2	Small
12 weeks post	=0.150	0.4 (±1.8)	0.2	Small	=0.265	0.3 (±1.6)	0.2	Small
4 weeks post								
8 weeks post	=0.539	0.1 (±0.8)	0.1		=0.083	0.3 (±1.1)	0.3	Small
12 weeks post	=0.722	−0.1 (±1.8)	0.1		=0.617	0.2 (±2.3)	0.1	
8 weeks post								
12 weeks post	=0.475	−0.2 (±1.5)	0.1		=0.695	−0.1 (±2.1)	0	

Further, the performance of the sealants was assessed (Table 6). Seventy-eight hypersensitive MIH molars were sealed in the upper jaw and 68 molars in the lower jaw. No significant differences were observed regarding the parameters evaluated (*p*> 0.05, Fisher test). In both materials, partial loss of the sealings occurred over 12 weeks. Within the resin-based group, this was the case after 8 weeks in one tooth. Regarding the glass ionomer group, this event was observed just after 1 week in one tooth increasing to eight sealings after 12 weeks. Also, these partial losses were minimal. Furthermore, in teeth sealed with Ketac Universal, deteriorations occurred largely in three domains. These were marginal integrity, and optical as well as tactile surface texture. The inter- and intra-examiner kappa values of the examiner were found to be higher than 0.80 in all cases. Discussion due to no complete agreement was necessary in some scores making the transition from “alfa” to “bravo.”

**Table 6:** Clinical scores of the restorations in both groups according to the modified Ryge criteria. No significant differences between groups were detected (*p*>0.05)

*N* = 39		Baseline			After 1 week			After 4 weeks			After 8 weeks			After 12 weeks			Total sealings evaluated
Score		A	B	C	A	B	C	A	B	C	A	B	C	A	B	C	
Resin-based sealant	Retention	39	0	0	39	0	0	39	0	0	37	1	0	37	1	0	38
	Marginal integrity	39	0	0	39	0	0	39	0	0	37	1	0	37	1	0	38
	Marginal discoloration	39	0	0	39	0	0	39	0	0	38	0	0	38	0	0	38
	Surface texture—optical	39	0	0	39	0	0	39	0	0	38	0	0	38	0	0	38
	Surface texture—tactile	39	0	0	39	0	0	39	0	0	38	0	0	38	0	0	38
	Caries	39	0	0	39	0	0	39	0	0	38	0	0	38	0	0	38
Glass ionomer	Retention	39	0	0	38	1	0	36	3	0	33	5	0	30	8	0	38
	Marginal integrity	39	0	0	37	2	0	29	10	0	26	12	0	22	16	0	38
	Marginal discoloration	39	0	0	39	0	0	39	0	0	38	0	0	38	0	0	38
	Surface texture—optical	39	0	0	39	0	0	21	18	0	10	28	0	8	30	0	38
	Surface texture—tactile	39	0	0	38	1	0	20	19	0	10	28	0	5	33	0	38
	Caries	39	0	0	39	0	0	39	0	0	38	0	0	38	0	0	38

## Discussion

This is the first study evaluating the effect of a sealing on hypersensitivity treatment of MIH-affected molars worldwide. Although some authors have already evaluated the desensitizing effect of different preventive approaches using CPP-ACP or arginine-based products [14, 15], effective management of hypersensitive MIH teeth is an ongoing issue for the majority of clinicians and researchers and studies on this topic are still scarce [10]. For the first time, the present clinical trial shows that a resin-based as well as a glass ionomer sealant reduced hypersensitivity in MIH-affected molars immediately and at least over 12 weeks.

It is well known that hypersensitivity is a subjective condition that is difficult to quantify. Evaporative stimuli and the Visual Analog Scale (VAS) are the most preferred methods to provoke and measure pain in patients with dentin hypersensitivity [30, 31] as pain intensity is higher for the air test compared to the tactile test [10, 15]. This approach was adopted in the present study. All included teeth were subjected to an air blast stimulus. However, instead of the VAS, the Wong-Baker Faces Scale was used. This is a common step in order to evaluate the intensity of the pain in children as this tool was originally created for children to help them communicate about their pain with facial expression drawings (“faces scales”) [26]. Although there is debate about the optimum design of the facial expressions, the literature suggests that they are the preferred method of pain reporting by children [32]. Previous studies focusing on the measurement of hypersensitivity of MIH-affected teeth also used this approach [14, 15].

Regarding the intensity of pain measured, our results showed that the mean value of the Wong-Baker Faces Scale score in both groups was 7.1. This underlines that the mean intensity of hypersensitivity was high in included patients. Interestingly, Raposo et al. [10] observed lower values in their study when focusing on the general prevalence of hypersensitivity in MIH molars, irrespectively of MIH severity. However, they also noted that cases of high-intensity hypersensitivity were observed, most of all represented by maximum scores.

As this is the first clinical trial to examine hypersensitivity before and after treatment of MIH molars with sealants at different time points, no direct comparisons can be made between current and previous data. Currently, there are only one to two studies focusing on the effect of CPP-ACP and arginine on hypersensitivity relief of MIH patients. Pasini et al. (2018) compared CPP-ACP and fluoride toothpaste [14]. In the CPP-ACP group, children were instructed to apply the paste with a tray for 2 h/day after toothbrushing. Sensitivity to thermal stimuli was evaluated at baseline and after 120 days after the beginning of the treatment. The sensitivity decreased significantly in the CPP-ACP group over 120 days while no differences were observed in the control group (1000-ppm fluoride toothpaste). Furthermore, Bekes et al. (2016) evaluated the application of an agent with 8% arginine and calcium carbonate over 8 weeks [15]. The authors were able to show that hypersensitivity could be reduced which was evident by a significant decrease in the SCASS scores which dropped from 2.1 (±0.3) to 0.8 (±0.9). Both of these studies were thus able to demonstrate that the prolonged use of pastes presents an approach to reduce hypersensitivity. In the present study, a different approach was taken. It was the aim to evaluate the effect of a single treatment on hypersensitivity relief in MIH-affected molars. Thereby, it was possible to show that SCASS and WBFS scores decreased significantly immediately after treatment regardless of the sealant material used. SCASS scores were reduced below a score of 1, which means that nearly all patients did no more respond to the air stimulus. This effect was stable over 12 weeks. Furthermore, the intensity of the pain could also be reduced to a large extend. Before treatment, all included teeth were at the higher end of the WBFS (7.0). After sealing, this score significantly decreased to 1.2 (±0.5; resin-based sealant) and 1.1 (±1.8; GIC), respectively. The current findings were also supported by a marked improvement in the patients’ overall OHRQoL [19]. As already shown in a recent publication [19], it was also found that the Child Perceptions Questionnaire (CPQ) score decreased significantly following treatment at the 1-week follow-up and was significantly reduced again after the 12-week follow-up. The biggest improvements were seen in the oral symptoms domain as well as the functional limitations, respectively.

The second aim of this study was to compare the retention rates of both materials used over 12 weeks. It was found that the resin-based material performed well. Only one sealant was partially lost after 8 weeks. In this special case, some problems occurred during sealant application in terms of compliance of the patient and keeping the tooth dry. In the glass ionomer group, a partial loss of retention was observed just after 1 week in one tooth increasing to eight teeth after 12 weeks. Furthermore, in teeth sealed with this material, deteriorations occurred largely in three domains. These were marginal integrity, and optical as well as surface texture.

To date, there are only less than a handful studies available evaluating the retention and different treatment protocols of sealants on MIH-affected teeth to which the present findings can be compared. Furthermore, these studies have only focused on resin-based sealants and beyond show conflicting evidence.

Kotsanos et al. [33] performed a retrospective study comparing sealed MIH molars with sound teeth. Sealants in MIH molars were found to need retreatment almost 2 years earlier than sealants in the control group and were three times more at failure risk compared to those on control teeth. In contrast, Fragelli et al. [34] discovered that molars affected by MIH presented a survival rate similar to the resin-based sealants placed in sound molars after 18-month follow-up. However, the authors also reported the loss of retention in three sealed MIH teeth (out of 25) within the first month. In the present study, one partial loss (out of 38) was observed after 8 weeks.

With respect to adhesive procedures which were used in case of the resin-based sealant in the present study, structural, mechanical, and chemical properties and changes of MIH-affected enamel compared to normal enamel must be considered. MIH-affected enamel is characterized by a reduction in mineral quantity and quality, a reduced hardness and modulus of elasticity as well as increased porosity and higher protein contents. Etched MIH-affected enamel also shows more cracks and deep pores than sound enamel; retentive etch patterns of MIH-affected enamel are suboptimal for subsequent bonding [35]. Nevertheless, until now, no satisfying procedure for stabilizing of MIH enamel is available [36]. As a result, cohesive failure is oftentimes noted in restorations bonded to MIH enamel [37]. Solid application protocols to improve adhesion to MIH-affected enamel and the longevity of restoration in these teeth have not been established yet and further research is needed [38].

Regarding different materials available for sealing, it is generally known that resin-based sealants present higher retention rates compared to glass ionomer cement sealants [20]. In the present study, partial losses of retention were observed in eight teeth. Although resin-based sealants are widely used in the prevention of caries in both MIH-affected and nonaffected molars, and seem to be preferable, it must be acknowledged that these materials need optimal conditions for placement, including excellent moisture control [39]. Vice versa, glass ionomer cement sealants can be placed in difficult clinical conditions as an interim treatment where isolation is inadequate as they are less technique sensitive, and rapidly set and do not require intermediate steps, such as etching. Especially in situations when an MIH molar has not fully erupted but a sealing is indicated as well as in non-cooperative, anxious children or patients suffering from hypersensitive and painful molars, glass ionomer cement sealants and their easy application procedure seem preferable and more convenient for the child. Therefore, glass ionomer cements may be used for temporization of teeth and might also provide some benefit via fluoride release [40]. Furthermore, glass ionomer sealants have recently proven to be effective in the prevention of caries lesions in MIH-affected molars after a follow-up period of 12 months [41]. The long-term retention of these restorations seems limited, probably as they have a low wear and fracture resistance [40, 42]. The lower survival rates have also been reported for glass ionomer cement restorations compared to composite restorations in MIH teeth that already show posteruptive breakdowns [21]. However, glass ionomer cement facilitates the mineralization process and protects the remaining structures from tooth caries lesion formation and tooth sensitivity [42].

With regard to treatment protocols, there is only one study available focusing on different application modes. Lygidakis et al. [18] analyzed sealant retention applied to MIH molars with occlusal enamel opacities, using two different application methods after 4 years. They found that with an adhesive agent being applied between the previously etched enamel surface and the sealant material, retention in MIH molars was substantially increased. The full retention rate after the 4-year period was 70.2% and no fissure sealing being totally lost. Based on these findings, a similar protocol for application of the resin-based sealant was chosen. In this study, a universal adhesive was applied prior to sealant application. No additional etching was done before adhesive application. It is known from clinical practice that children with hypersensitive molars in most cases do not tolerate the acid on the affected tooth without anesthesia as it provokes pain leading to reduced cooperation. In exchange, it was acknowledged that sealants applied in this mode of action do not achieve the same retention rates as conventionally applied sealants [20]. However, the focus was the implementation of a simple and clinically feasible mode and not to lose the children’s compliance.

The presents study shows some limitations. Firstly, one major limitation is that the projected sample size could not be reached. Both study centers were only able to include 39 patients during a time frame of 2 years. This was the result of applying rigorous inclusion criteria that allowed only hypersensitive MIH molars with no sign of posteruptive breakdown at any surface being included. Thus, the study was terminated before reaching the targeted sample size. This might have affected the interpretation of our results. However, all included patients showed similar results regarding the significant decrease in hypersensitivity. We assume that the missing patients would have experienced improvements likewise. Another limitation of this study includes the subjective nature of hypersensitivity assessment and the challenge to quantify the pain intensity generated by stimuli. In this respect, the faces scale was used as these represent the most popular method when it comes to pediatric samples [43]. Additionally, no untreated controls were used, which might have affected the interpretation of our results. However, we considered it unethical to have untreated controls. Moreover, due to the lack of studies that used a similar methodology, the comparison of our data was not possible. On the other hand, it should be stressed that this is the first clinical study that assessed dental hypersensitivity associated with MIH through validated tests and scales, which can bring some light into the topic, as hypersensitivity is frequently reported as one of the major clinical challenges related to the clinical management of MIH.

## Conclusion

In conclusion, both materials (composite sealant and glass ionomer) were able to reduce hypersensitivity successfully immediately and throughout the 12-week follow-up. Furthermore, both sealant materials performed similar in terms of retention.

## References

[1] Weerheijm KL, Jalevik B, Alaluusua S (2001). Molar-incisor hypomineralisation. Caries Res.

[2] Schwendicke F, Elhennawy K, Reda S, Bekes K, Manton DJ, Krois J (2018). Global burden of molar incisor hypomineralization. J Dent.

[3] Silva MJ, Scurrah KJ, Craig JM, Manton DJ, Kilpatrick N (2016). Etiology of molar incisor hypomineralization - a systematic review. Community Dent Oral Epidemiol.

[4] Weerheijm KL (2003). Molar incisor hypomineralisation (MIH). Eur J Paediatr Dent.

[5] Jalevik B, Noren JG (2000). Enamel hypomineralization of permanent first molars: a morphological study and survey of possible aetiological factors. Int J Paediatr Dent.

[6] Lygidakis NA (2010). Treatment modalities in children with teeth affected by molar-incisor enamel hypomineralisation (MIH): a systematic review. Eur Arch Paediatr Dent.

[7] Jalevik B, Klingberg GA (2002). Dental treatment, dental fear and behaviour management problems in children with severe enamel hypomineralization of their permanent first molars. Int J Paediatr Dent.

[8] Fagrell T (2011). Molar incisor hypomineralization. Morphological and chemical aspects, onset and possible etiological factors. Swed Dent J Suppl.

[9] Rodd HD, Boissonade FM, Day PF (2007). Pulpal status of hypomineralized permanent molars. Pediatr Dent.

[10] Raposo F, de Carvalho Rodrigues AC, Lia EN, Leal SC (2019). Prevalence of hypersensitivity in teeth affected by molar-incisor hypomineralization (MIH). Caries Res.

[11] Lygidakis NA, Wong F, Jalevik B, Vierrou AM, Alaluusua S, Espelid I (2010). Best Clinical Practice Guidance for clinicians dealing with children presenting with molar-incisor-hypomineralisation (MIH): an EAPD Policy Document. Eur Arch Paediatr Dent.

[12] Ghanim A, Silva MJ, Elfrink MEC, Lygidakis NA, Marino RJ, Weerheijm KL, Manton DJ (2017). Molar incisor hypomineralisation (MIH) training manual for clinical field surveys and practice. Eur Arch Paediatr Dent.

[13] Baroni C, Marchionni S (2011). MIH supplementation strategies: prospective clinical and laboratory trial. J Dent Res.

[14] Pasini M, Giuca MR, Scatena M, Gatto R, Caruso S (2018). Molar incisor hypomineralization treatment with casein phosphopeptide and amorphous calcium phosphate in children. Minerva Stomatol.

[15] Bekes K, Heinzelmann K, Lettner S, Schaller HG (2017). Efficacy of desensitizing products containing 8% arginine and calcium carbonate for hypersensitivity relief in MIH-affected molars: an 8-week clinical study. Clin Oral Investig.

[16] Ahovuo-Saloranta A, Forss H, Walsh T, Nordblad A, Makela M, Worthington HV (2017). Pit and fissure sealants for preventing dental decay in permanent teeth. Cochrane Database Syst Rev.

[17] Llodra JC, Bravo M, Delgado-Rodriguez M, Baca P, Galvez R (1993). Factors influencing the effectiveness of sealants: a meta-analysis. Community Dent Oral Epidemiol.

[18] Lygidakis NA, Dimou G, Stamataki E (2009). Retention of fissure sealants using two different methods of application in teeth with hypomineralised molars (MIH): a 4 year clinical study. Eur Arch Paediatr Dent.

[19] Bekes K, Amend S, Priller J, Zamek C, Stamm T, Kramer N (2021) Changes in oral health-related quality of life after treatment of hypersensitive molar incisor hypomineralization-affected molars with a sealing. Clin Oral Investig. 10.1007/s00784-021-03947-z 10.1007/s00784-021-03947-z PMC8531117 33876317

[20] Kuhnisch J, Bedir A, Lo YF, Kessler A, Lang T, Mansmann U, Heinrich-Weltzien R, Hickel R (2020). Meta-analysis of the longevity of commonly used pit and fissure sealant materials. Dent Mater.

[21] Linner T, Khazaei Y, Bucher K, Pfisterer J, Hickel R, Kuhnisch J (2020). Comparison of four different treatment strategies in teeth with molar-incisor hypomineralization-related enamel breakdown-a retrospective cohort study. Int J Paediatr Dent.

[22] Weerheijm KL, Duggal M, Mejare I, Papagiannoulis L, Koch G, Martens LC, Hallonsten AL (2003). Judgement criteria for molar incisor hypomineralisation (MIH) in epidemiologic studies: a summary of the European meeting on MIH held in Athens, 2003. Eur J Paediatr Dent.

[23] Schiff T, Delgado E, Zhang YP, Cummins D, DeVizio W, Mateo LR (2009) Clinical evaluation of the efficacy of an in-office desensitizing paste containing 8% arginine and calcium carbonate in providing instant and lasting relief of dentin hypersensitivity. Am J Dent 22 Spec No A:8A-15A. 19472556

[24] Bekes K, Steffen R (2016). The Wuerzburg MIH concept: Part 1. The MIH treatment need index (MIH TNI). A new index to assess and plan the treatment in patients with molar incisor hypomineralization (MIH). Oralprophylaxe & Kinderzahnheilkunde.

[25] Steffen R, Kramer N, Bekes K (2017). The Wurzburg MIH concept: the MIH treatment need index (MIH TNI) : a new index to assess and plan treatment in patients with molar incisor hypomineralisation (MIH). Eur Arch Paediatr Dent.

[26] Wong DL, Baker CM (1988). Pain in children: comparison of assessment scales. Pediatr Nurs.

[27] Ryge G (1980). Clinical criteria. Int Dent J.

[28] Goldberg AJ, Rydinge E, Santucci EA, Racz WB (1984). Clinical evaluation methods for posterior composite restorations. J Dent Res.

[29] Dukic W, Glavina D (2007). Clinical evaluation of three fissure sealants: 24 month follow-up. Eur Arch Paediatr Dent.

[30] Holland GR, Narhi MN, Addy M, Gangarosa L, Orchardson R (1997). Guidelines for the design and conduct of clinical trials on dentine hypersensitivity. J Clin Periodontol.

[31] Bijur PE, Silver W, Gallagher EJ (2001). Reliability of the visual analog scale for measurement of acute pain. Acad Emerg Med.

[32] Keck JF, Gerkensmeyer JE, Joyce BA, Schade JG (1996). Reliability and validity of the Faces and Word Descriptor Scales to measure procedural pain. J Pediatr Nurs.

[33] Kotsanos N, Kaklamanos EG, Arapostathis K (2005). Treatment management of first permanent molars in children with molar-incisor hypomineralisation. Eur J Paediatr Dent.

[34] Fragelli CMB, Souza JF, Bussaneli DG, Jeremias F, Santos-Pinto LD, Cordeiro RCL (2017). Survival of sealants in molars affected by molar-incisor hypomineralization: 18-month follow-up. Braz Oral Res.

[35] Elhennawy K, Manton DJ, Crombie F, Zaslansky P, Radlanski RJ, Jost-Brinkmann PG, Schwendicke F (2017). Structural, mechanical and chemical evaluation of molar-incisor hypomineralization-affected enamel: a systematic review. Arch Oral Biol.

[36] Kramer N, Bui Khac NN, Lucker S, Stachniss V, Frankenberger R (2018). Bonding strategies for MIH-affected enamel and dentin. Dent Mater.

[37] William V, Burrow MF, Palamara JE, Messer LB (2006). Microshear bond strength of resin composite to teeth affected by molar hypomineralization using 2 adhesive systems. Pediatr Dent.

[38] Lagarde M, Vennat E, Attal JP, Dursun E (2020). Strategies to optimize bonding of adhesive materials to molar-incisor hypomineralization-affected enamel: a systematic review. Int J Paediatr Dent.

[39] Cvikl B, Moritz A, Bekes K (2018) Pit and fissure sealants-a comprehensive review. Dent J (Basel) 6. 10.3390/dj6020018 10.3390/dj6020018 PMC6023524 29895726

[40] Elhennawy K, Schwendicke F (2016). Managing molar-incisor hypomineralization: a systematic review. J Dent.

[41] Schraverus MS, Olegario IC, Bonifacio CC, Gonzalez APR, Pedroza M, Hesse D (2021) Glass ionomer sealants can prevent dental caries but cannot prevent posteruptive breakdown on molars affected by molar incisor hypomineralization: one-year results of a randomized clinical trial. Caries Res 1-9. 10.1159/000516266 10.1159/000516266 PMC8491481 34107492

[42] Fragelli CM, Souza JF, Jeremias F, Cordeiro Rde C, Santos-Pinto L (2015) Molar incisor hypomineralization (MIH): conservative treatment management to restore affected teeth. Braz Oral Res 29. 10.1590/1807-3107BOR-2015.vol29.0076 10.1590/1807-3107BOR-2015.vol29.0076 26083091

[43] Chambers CT, Giesbrecht K, Craig KD, Bennett SM, Huntsman E (1999). A comparison of faces scales for the measurement of pediatric pain: children's and parents' ratings. Pain.

